# Factorial Validity of the German KABC-II at Ages 7 to 12 in a Clinical Sample: Four Factors Fit Better than Five

**DOI:** 10.3390/jintelligence11070148

**Published:** 2023-07-22

**Authors:** Gerolf Renner, Anne Schroeder, Dieter Irblich

**Affiliations:** 1 Faculty of Special Education, Ludwigsburg University of Education, 71634 Ludwigsburg, Germany; 2 Werner Otto Institute, 22337 Hamburg, Germany; 3 Social Pediatric Center Kreuznacher Diakonie, 55469 Simmern, Germany

**Keywords:** Kaufman Assessment Battery for Children—Second Edition, KABC-II, confirmatory factor analysis, assessment, intelligence test, factorial validity, construct validity, Social Pediatric Center

## Abstract

Multidimensional intelligence test batteries such as the KABC-II are widely used in clinical practice. Although validity evidence should be provided for all intended uses of a test, data on the factorial validity of the KABC-II mostly relies on the standardization samples and raises some concerns about the adequacy of the factor structure. Confirmatory factor analyses of the KABC-II core subtests were conducted in a sample of 627 children who had been assessed in German Centers for Social Pediatrics. The standard structure of the KABC-II was superior to unidimensional models but, as in previous research, evidenced cross-loadings and a high correlation between *Planning/Gf* and *Simultaneous/Gv*. *Pattern Reasoning* was more closely related to *Simultaneous/Gv* than to *Planning/Gf*. A four-factorial structure combining subtests from *Planning/Gf* and *Simultaneous/Gv* to form a common factor emerged as a better representation of the data. *Story Completion* showed a secondary loading on *Knowledge/Gc*. On average, most subtest variance was accounted for by the general factor. Models with bonus points for fast responses generally fitted worse than those without. Clinicians should be aware that *Planning/Gf* and *Simultaneous/Gv* measure both visual and fluid abilities. Scales of the KABC-II should not be interpreted as dimensions independent of the general factor.

## 1. Introduction

Clinical use and interpretation of standardized assessment instruments needs to be informed by scientific evidence. One of the quality criteria to be met by a standardized test is the factorial validity. It refers to the extent to which the putative structure of a test is supported by empirical data (American Educational Research Association et al. 2014) and is an important precondition for the interpretation of test results. When tests lack factorial validity, scales cannot be interpreted as measuring the constructs they are supposed to measure. If, for example, subtests are empirically related to several scales, test results may be influenced both by the specific construct that is suggested by the name of a scale and by other abilities.

### 1.1. Theoretical Background and Structure of the KABC-II

The Kaufman Assessment Battery for Children—Second Edition (Kaufman and Kaufman 2004a; see also Kaufman et al. 2005) is a multidimensional measure of cognitive abilities for children and adolescents in the age range of 3 to 18 years. The purpose of the KABC-II is to contribute to “psychological, clinical, psychoeducational, and neuropsychological evaluations” (Kaufman and Kaufman 2004a, p. 8) and to inform clinical diagnoses, treatment planning, and placement decisions. These are high-stakes applications that require comprehensive validity evidence.

The German adaptation of the KABC-II (Melchers and Melchers 2015) is widely used in clinical settings (Irblich et al. 2020) and special education (Joél 2021). This study focuses on the clinical application of the KABC-II at ages 7 to 12 in five German Social Pediatric Centers (SPCs). SPCs provide multidisciplinary assessment and intervention for children and adolescents with disabilities, developmental and psychiatric disorders, and chronic illnesses (Ehrich et al. 2016).

The KABC-II claims to be founded on two theoretical models: the Cattell–Horn–Carroll (CHC) theory of intelligence (McGrew 1997; Flanagan and Ortiz 2001), and Luria’s (1966) neuropsychological theory of cognitive processing.

Based on factor-analytic studies, CHC theory seeks to provide a comprehensive taxonomy of cognitive abilities, organized in three strata with varying degrees of generality: “narrow” abilities (stratum I), “broad” abilities (stratum II), and general intelligence, corresponding to the g-factor (stratum III). In its latest version, Schneider and McGrew (2018) identify 18 broad abilities, each consisting of several narrow abilities. The subtest structure of the KABC-II changes across age groups. At ages 7 to 12, the KABC-II is composed of 10 core and 6 supplementary subtests. The core subtests are grouped into five scales: *Fluid Reasoning (Gf)*, *Visual Processing (Gv)*, *Crystallized Ability (Gc)*, *Short-Term Memory (Gsm)*, and *Long-Term Storage and Retrieval (Glr)*, corresponding to eponymous CHC factors. However, this structure does not consequently align with CHC theory. As classified by Kaufman and Kaufman (2004a) and Flanagan et al. (2013), several subtests (e.g., *Rover*, *Story Completion*) are intended to measure narrow abilities that are subsumed under different factors on stratum II (Table 1). Consequently, the scales *Fluid reasoning (Gf)*, *Visual Processing (Gv)*, and *Crystallized Ability (Gc)* are actually related to two or more broad abilities.

When using the Luria model, subtests of *Crystallized Ability (Gc)* are not administered. The assignment of subtests to the remaining scales is identical. In the Luria model, the scales are termed *Sequential Processing*, *Simultaneous Processing*, *Learning Ability*, and *Planning Ability*. Thus, the Luria model is just a CHC model without Gc, although its aim is to measure different constructs. In the following text, we will use the common terminology employed in the manual: *Planning/Gf*, *Simultaneous/Gv*, *Knowledge/Gc*, *Sequential/Gsm*, and *Learning/Glr*.

All core subtests equally contribute to global scales, termed the *Fluid-Crystallized Index* (FCI; CHC model) and the *Mental Processing Index* (MPI; Luria model). Supplementary subtests may replace core subtests according to the rules provided in the manual or contribute to a more comprehensive measurement of the constructs that are of interest. At ages 7 to 12, three core subtests (*Triangles*, *Story Completion*, *Rover*) have a time limit. On three subtests (*Triangles*, *Pattern Reasoning*, *Story Completion*), the standard scoring procedure credits rapid correct responses with extra points. However, test users have the option to score these subtests based on correct responses only. Time points were introduced because scoring without time points “has the disadvantage that it does not differentiate among higher-ability adolescents” (Kaufman and Kaufman 2004a, p. 26).

When evaluating the structure of the KABC-II, there is a need to know whether the scales intend to measure distinct constructs or a blend of specific and general abilities. In confirmatory factor analyses (CFA), the former interpretation is best mirrored by a bifactor model (e.g., Watkins and Beaujean 2014), and the latter by a higher-order model. In bifactor models, all subtests are allowed to load directly on a general factor. Variance not accounted for by the general factor is captured by uncorrelated group factors. Thus, group factors are defined by the shared variance between a set of subtests once the influence of the general factor has been partitioned out. In bifactor models, subtest scores are directly influenced by the general factor, whereas this influence is mediated by first-order factors in higher-order models (Keith and Reynolds 2018; Markon 2019).

Kaufman and Kaufman (2004a) propose a multistage interpretation procedure that aims at identifying inter- and intra-individual strengths and weaknesses. In this process, broad abilities are intended to be “of primary importance for interpreting the child’s cognitive profile” (Kaufman and Kaufman 2004a, p. 16). FCI and MPI are considered as “almost always secondary in importance to fluctuations within the scale profile” (Kaufman and Kaufman 2004a, p. 43). With these aims in mind, we would expect test construction to focus on the development of subtests and scales that are strong and uncontaminated indicators of the constructs measured. However, Kaufman and Kaufman (2004a) did not advocate the development of pure measures of CHC broad abilities: “… the goal of comprehensive tests of cognitive ability like the KABC-II is to measure problem solving in different contexts and under different conditions, with complexity being necessary to assess high-level functioning. Toward that clinical goal, the authors strove to construct measures that featured a particular ability while incorporating aspects of other abilities” (Kaufman and Kaufman 2004a, p. 16). Thus, at least some subtests were constructed to reflect multiple abilities, but scales are interpreted as indicators of specific constructs.

### 1.2. Confirmatory Factor Analyses of the CHC Test Structure at Ages 7 to 12

The first data on the factorial validity of the KABC-II at ages 7 to 12 were presented by Kaufman and Kaufman (2004a). A higher-order model of core subtests corresponding to the test structure was evaluated by CFA. The model was supported by global fit indices. However, a standardized path coefficient of 1.01 between g and *Planning/Gf* revealed an inadmissible solution, probably a Heywood case. Inadmissible solutions may indicate misspecification and are considered untrustworthy (Kline 2016). Nevertheless, the results were interpreted as an “extremely good fit to the data” (Kaufman and Kaufman 2004a, p. 105). Average variance extracted (AVE, calculated on the basis of the factor loadings provided in the manual) was low for *Planning/Gf* (0.42) and *Simultaneous/Gv* (0.42), indicating low convergent validity of the subtests. No alternative models were tested.

An analogous CFA reported in the German manual of the KABC-II (Melchers and Melchers 2015) showed an adequate fit. Again, rival models were not tested. AVE was lowest for *Planning/Gf* (0.37) and *Simultaneous/Gv* (0.39). The loading of *Planning/Gf* on g was close to unity, indicating redundancy of these factors. In summary, the data reported in both manuals indicate that the standard test structure lacks sufficient support for ages 7 to 12.

Most subsequent CFA utilized the US standardization sample of the KABC-II. The analyses differed in age ranges studied, including supplemental subtests, and allowing various types of correlated errors or cross-loadings. Surprisingly, most studies did not investigate the standard test structure with 10 core subtests, which is most relevant for clinical use and interpretation of the KABC-II.

In an important exception, McGill (2020) conducted a reanalysis of the KABC-II normative update (KABC-II NU; Kaufman and Kaufman 2018). The KABC-II NU provides updated norms, while the content and structure of the test did not change. At ages 7 to 12, the sample comprised 250 participants. Confirmatory factor analyses were conducted for various higher-order, hierarchical, and bifactor models. Fit statistics demonstrated the superiority of a four-factor hierarchical model, with subtests of *Planning/Gf* and *Simultaneous/Gv* forming a common factor. In the standard model, *Planning/Gf* and *Simultaneous/Gv* were highly intercorrelated (0.92), indicating that they were almost indistinguishable.

Based on normative data of the KABC-II, McGill (2017) proposed an alternative structure for the standard Luria model with eight subtests, permitting *Pattern Reasoning* to load on both *Planning/Gf* and *Simultaneous/Gv*.

Reynolds et al. (2007) included supplemental subtests in a CFA of a KABC-II standardization sample for ages 6 to 18. They reproduced the Heywood case reported in the manual for a model based on subtest configurations proposed by the publishers. Their final model included a cross-loading of *Pattern Reasoning* on *Simultaneous/Gv* and loadings on additional factors of two supplemental tests (*Gestalt Closure* on *Knowledge/Gc*, *Hand Movements* on *Planning/Gf*). In a similar model (Benson et al. 2016), five-factorial solutions were not admissible due to the negative error variance of *Planning/Gf*. Both the four-factorial higher-order model (allowing cross-loadings, e.g., *Pattern Reasoning* on *Simultaneous/Gv*, and direct paths from the second-order factor to *Pattern Reasoning* and *Story Completion*) and the bifactor model fit the data well. Similar to Reynolds et al. (2007), they found that models without time points fit better than those with time points.

Other studies focused on research questions, such as a prediction of achievement, and included supplementary subtests or additional measures, mostly based on the co-normed standardization sample data of the KABC-II and the Kaufman Test of Educational Achievement, Second Edition (KTEA II; Kaufman and Kaufman 2004b). Final higher-order models of Kaufman et al. (2012) and Villeneuve et al. (2019) allowed for cross-loadings, as proposed by Reynolds et al. (2007), including *Pattern Reasoning* on *Simultaneous/Gv*. In the final model of Villeneuve et al. (2019), *Planning/Gf* was not distinguishable from the general factor.

So far, studies on the factorial validity of the KABC-II in independent samples are scarce (e.g., Malda et al. 2010; Mitchell et al. 2018), and they were conducted with major modifications of the test structure.

In summary, alternative CFA models, notably those allowing *Pattern Reasoning* to load on *Simultaneous/Gv*, were superior to the standard test structure in most studies (at ages 5 and 6, *Pattern Reasoning* is a subtest of *Simultaneous/Gv*). Some results question separating *Planning/Gf* and *Simultaneous/Gv* and show that *Planning/Gf* is almost identical to the general factor. The difficulty of differentiating Gf and Gv has also been noted in several CFAs (e.g., Canivez et al. 2020; Dombrowski et al. 2018; Lecerf and Canivez 2018; Pauls and Daseking 2021) of the Wechsler Intelligence Scale for Children—Fifth Edition (WISC-V; Wechsler 2014). Although the use of time points is advocated in the manual of the KABC-II, models based on subtests without time points are more closely aligned with the test structure.

### 1.3. Purpose

The present study endeavors to make the following contributions: (1) To extend our knowledge of the factorial structure of the KABC-II at ages 7 to 12 by using CFA of g-factor, second-order, and bifactor models, including modifications based on CHC theory. (2) To provide the first independent data on the factor structure in a clinical sample of children with heterogeneous developmental disorders. So far, no study on the psychometric properties of the KABC-II has been conducted in applied clinical settings. As demanded by the Standards for Educational and Psychological Testing (American Educational Research Association et al. 2014), validity evidence should be provided for all intended uses of a test. When testing children with psychiatric and developmental disorders or disabilities, deficits in attention and self-regulation, limitations in access skills (e.g., motor impairment), test anxiety, etc., may compromise the validity of the test results. Therefore, psychometric data that rely only on standardization samples should be complemented by clinical studies.

## 2. Materials and Methods

### 2.1. Participants

The participants were 627 children, aged 7 to 12, that had been assessed between April 2015 and October 2021 due to various developmental, behavioral, or emotional disorders in 5 SPCs in southwest (Simmern), north (Hamburg, Bremerhaven), and northeast (Berlin, Rostock) Germany. Standards of assessment in SPCs are described by Hollmann et al. (2014). All assessments were conducted by experienced clinical psychologists, adhering to the rules for test administration and scoring described in the German manual.

Standard scores for subtests and scales of the KABC-II, various demographic variables, and diagnoses according to ICD-10 were extracted from clinical records. Detailed information on the participant characteristics is provided in Table 2. Test protocols were included only when children had been tested with all core subtests.

### 2.2. Instrument

The German adaptation of the KABC-II (Melchers and Melchers 2015) closely follows the structure and content of the original test. Norms were collected from April 2013 through February 2014. The total norming sample comprised 1745 children, including 656 participants aged 7 to 12. Descriptions of the KABC-II subtest are available in the test manuals (Kaufman and Kaufman 2004a; Melchers and Melchers 2015) and in Kaufman et al.’s work (2005).

### 2.3. Statistical Analyses and Models

AMOS 28 (Arbuckle 2021) was used to conduct CFA with maximum likelihood estimation based on age-referenced subtest scores. We first tested a series of models (see Table 3) based on all core subtests with timed scores for *Triangles*, *Story Completion*, and *Pattern Reasoning*:

- Model 1: A first-order model with all core subtests loading on a single-factor (g-factor). To achieve identifiability, one subtest loading was fixed to one.
- Model 2: A second-order (three-stratum) model reflecting the standard test structure with one second-order factor and five first-order factors. One loading of each factor was fixed to one. Model 2 was used as a baseline model for comparisons with modified models allowing cross-loadings of subtests. These models were selected based on the CHC narrow-ability classifications (Table 1) and previous research:
  ○ 2a: *Riddles* allowed to load on *Planning/Gf*
  ○ 2b: *Story Completion* allowed to load on *Knowledge/Gc*
  ○ 2c: *Story Completion* allowed to load on *Simultaneous/Gv*
  ○ 2d: *Rover* allowed to load on *Planning/Gf*
  ○ 2e: *Pattern Reasoning* allowed to load on *Simultaneous/Gv*
  ○ 2f: A model including all significant cross-loadings from models 2a to 2e
- Model 3: A bifactor model with all subtests loading on a general factor and five orthogonal group factors corresponding to the scales of the KABC-II. To achieve identifiability, loadings of all subtests on group factors and of one subtest on the general factor were fixed to one.
- Model 4: A four-factor, second-order model, combining subtests of *Simultaneous/Gv* and *Planning/Gf* to form a single factor. Variants 4a and 4b included modifications as specified in models 2a and 2b. Model 4 is identical to the final model chosen by McGill (2020).
- Model 5: A four-factor bifactor model, with four group factors, including a combined Gf/Gv factor.

Furthermore, the effects of substituting timed scores with untimed scores of *Triangles*, *Story Completion*, and *Pattern Reasoning* were investigated for the standard model and selected models of the preceding analyses.

Univariate normality was assumed for skewness < 2 and kurtosis < 7 (West et al. 1995). Multivariate normality was assessed by Mardia’s coefficient. SPSS 27 (IBM Corp 2020) was used for descriptive analyses. Scaled scores were compared with standardization data by one-sample *t*-tests. Cohens *d* was calculated as a measure of the effect size.

As recommended by Kline (2016), the following indices were used along with the χ^2^ test to assess model fit: the comparative fit index (CFI), the root mean square error of approximation (RMSEA), the standardized root mean square residual (SRMR), and the Akaike information criterion (AIC). Adequate model fit was assumed with CFI ≥ 0.95, SRMR ≤ 0.05, and RMSEA ≤ 0.06 (Hu and Bentler 1999; Schermelleh-Engel et al. 2003). Model comparisons were evaluated by χ^2^ difference tests for nested models. Additionally, differences in AIC (ΔAIC) and Akaike weights (Wagenmakers and Farrell 2004) were calculated. ΔAIC is the difference between the minimal AIC of the models considered and the AIC for a given model. For the best-fitting model, ΔAIC will be zero. According to Burnham and Anderson (2004), models with ΔAIC ≤ 2 have substantial support, models with 4 ≤ ΔAIC ≤ 7 have considerably less support, and models with ΔAIC ≥ 10 have no support. Akaike weights (*w_i_* AIC) can be interpreted as the probability that a model is the best of several models considering the data.

According to Kline (2016), models should never be retained “based solely on global fit testing” (p. 461). Therefore, the presence of local fit problems (e.g., negative variances, non-significant factor loadings) was evaluated in all models. Coefficient omega (ω) and average variance extracted (AVE) will be reported for selected models of interest. AVE allows assessing the convergent validity of subtests of a scale, while omega estimates the proportion of variance in the observed scores explained by a common latent variable. AVE ≥ 0.50 and ω ≥ 0.70 will be considered adequate. For second-order models, proportions of subtest variance accounted for by the general factor, second-order, and uniqueness were computed, as outlined by Brunner et al. (2012).

Models with cross-loadings were considered only (a) when global fit was superior to the respective model without cross-loadings and (b) when cross-loadings were statistically significant.

For bifactor models, explained common variance (ECV) and omega were computed for the general factor (omega hierarchical, ω_H_) and the group factors (omega hierarchical subscale, ω_HS_) using the Omega program (Watkins 2013). For ω_H_, Reise et al. (2013) proposed a minimum value of 0.50. Higher ECV values indicate a stronger general factor (Reise et al. 2010).

## 3. Results

### 3.1. Preliminary Analyses

Descriptive statistics of scales and subtests are displayed in Table S1. Skewness and kurtosis of all subtests fell into the acceptable range proposed by West et al. (1995). Mardia’s coefficient of multivariate kurtosis was 4.70 and significantly differed from zero (critical ratio 4.18). Therefore, the Bollen–Stine bootstrap method (Bollen and Stine 1992) with 2000 bootstrap samples (Nevitt and Hancock 2001) was used to correct for potential biases of the χ^2^ statistic.

As expected, in a clinical sample, the subtest, scales, and global scores were significantly lower compared to normative data. One-sample *t*-tests showed a large effect for the FCI (*t*(626) = −20.54, *p* < 0.001, *d* = −0.82) and the MPI (*t*(626) = −21.41, *p* < 0.001, *d* = −0.95). Intercorrelations of the subtests are provided in Table S2.

### 3.2. Confirmatory Factor Analyses of Core Subtests (With Time Points)

Global fit statistics for all models are shown in Table 4.

Unidimensional model: Global fit was clearly inadequate according to RMSEA, SRMR, and CFI. The model was inferior to all other models according to χ^2^ difference tests for nested models (*p* < 0.001) and ΔAIC (≥491.08). Loadings of subtests on the general factor are displayed in Table 5.

**Figure 1:**
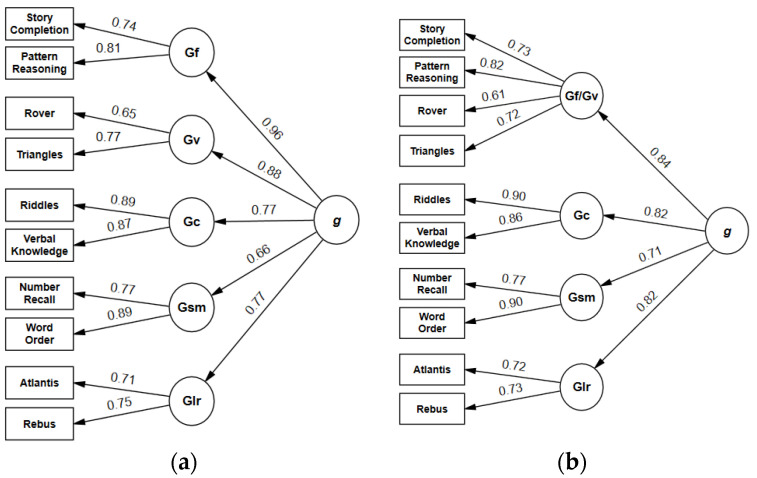
(**a**) Second-order model 2 of KABC-II core subtests with time points. χ^2^ = 106.17, *df* = 30, *p* < 0.001, CFI = 0.973, RMSEA = 0.064, SRMR = 0.38, AIC = 156.166. (**b**) Second-order model 4 of KABC-II core subtests with time points. χ^2^ = 72.87, *df* = 31, *p* < 0.001, CFI = 0.985, RMSEA = 0.046, SRMR = 0.029.

**Figure 2:**
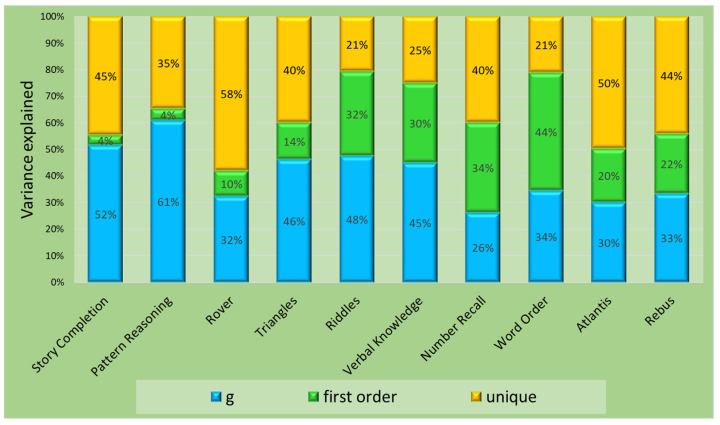
Sources of variance for the KABC-II core subtests. Values are based on model 2.

Five-factorial second-order models: Model 2, corresponding to the standard test structure and thus of special interest, was not fully adequate due to the RMSEA (0.064) slightly exceeding the cutoff value. CFI and SRMR fell within the acceptable ranges. All regression coefficients (Figure 1) were statistically significant. Loadings of first-order factors on the second-order factor ranged from 0.66 (*Sequential/Gsm*) to 0.96 (*Planning/Gf*). The partitioning of variance did not yield a consistent pattern (Figure 2). The general factor explained 26% (*Number Recall*) to 61% (*Pattern Reasoning*) of the subtest variance. Broad abilities accounted for an additional 4% (*Pattern Reasoning*) to 44% (*Word Order*), and unique variance ranged from 21% (*Riddles*) to 58% (*Rover*). AVE was greater than 0.50 for all scales, and omega surpassed the threshold of 0.70 for *Sequential/Gsm*, *Planning/Gf*, and *Knowledge/Gc* (Table 6). Implied correlations of first-order factors ranged from 0.51 to 0.85 (Table S3).

Model 2 served as a baseline model for comparisons with the modified second-order models. Inadmissible solutions were found for models 2a (loading of *Riddles* on *Knowledge/Gc* > 1.0) and 2d (negative error variance of *Triangles*, indicating a Heywood case). Therefore, these models were not considered further. CFI and SRMR were adequate for all models. RMSEA fell above the specified cutoff value for all models except 2e and 2f.

For Model 2b, the χ^2^ difference test (Δχ^2^(1) = 8.293, *p* = 0.004) suggested an improved fit. *Story Completion* significantly loaded on *Knowledge/Gc* (λ = 0.19, *p* = 0.002). In model 2c (Δχ^2^(1) = 0.014, *p* = 0.906), the cross-loading of *Story Completion* on *Simultaneous/Gv* was not significant (λ = −0.02, *p* = 0.910). Models 2e (Δχ^2^(1) = 41.67, *p* < 0.001) and 2f (Δχ^2^(2) = 43.78, *p* < 0.001) were significantly superior to the baseline model. In both models, *Pattern Reasoning* loaded stronger on *Simultaneous/Gv* (2e: λ = 0.55, *p* < 0.001; 2f: λ = 0.54, *p* < 0.001) than on *Planning/Gf* (2e: λ = 0.32, *p* = 0.003; 2f: λ = 0.35, *p* = 0.005). In model 2f, the path from *Story Completion* to *Knowledge/Gc* was not significant (λ = 0.12, *p* = 0.123).

Comparing all five-factorial second-order models with ΔAIC and Akaike weights showed that models 2e (ΔAIC = 0.11, *w_i_* AIC = 0.49) and 2f (ΔAIC = 0.00, *w_i_* AIC = 0.51) represented the data equally well. Due to the non-significant cross-loading of *Story Completion* in 2f, model 2e was considered preferable.

**Figure 3:**
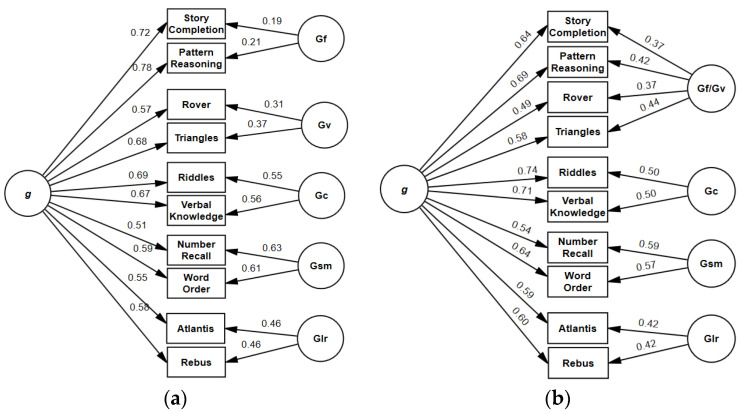
(**a**) Bifactor model 3 of KABC-II core subtests. χ^2^ = 64.50, *df* = 29, *p* < 0.001, CFI = 0.973, RMSEA = 0.064, SRMR = 0.038. (**b**) Bifactor model 5 of KABC-II core subtests. χ^2^ = 66.48, *df* = 31, *p* = 0.003, CFI = 0.987, RMSEA = 0.043, SRMR = 0.026.

Five-factorial bifactor model: For model 3 (Figure 3), all fit indices were identical to model 2 (Table 4). Loadings of subtests on the general factor ranged from 0.51 (*Number Recall*) to 0.78 (*Pattern Reasoning*). All subtest loadings on the general factor and group factors were significant. ECV of group factors ranged from 0.01 (*Planning/Gf*) to 0.13 (*Sequential/Gsm*). The ω_H_ coefficient for the general factor was high (0.83), whereas ω_HS_ for all group factors, ranging from 0.05 (*Planning/Gf*) to 0.46 (*Sequential/Gsm*), fell below the specified criterion (Table 7).

Four-factorial second-order models: All four-factorial models showed an adequate fit according to CFI, SRMR, and RMSEA (Table 4). Model 4b emerged as the best of these models according to χ^2^ difference tests, ΔAIC, and Akaike weights (*w_i_* AIC = 1.00). The path from *Knowledge/Gc* to *Story Completion* was significant (λ = 0.24, *p* < 0.001).

**Figure 4:**
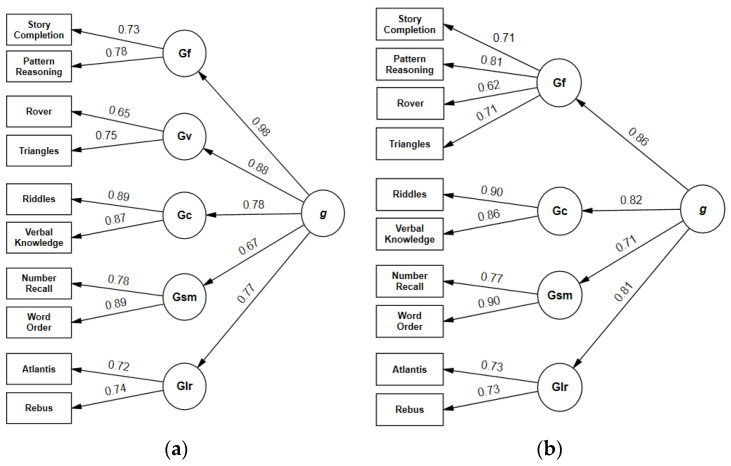
(**a**) Second-order model 2 of KABC-II core subtests without time points. χ^2^ = 90.50, *df* = 30, *p* < 0.001, CFI = 0.978, RMSEA = 0.057, SRMR = 0.035. (**b**) Second-order model 4 of KABC-II core subtests without time points. χ^2^ = 61.81, *df* = 31, *p* = 0.001, CFI = 0.989, RMSEA = 0.040, SRMR = 0.028.

Four-factorial bifactor model: CFI, RMSEA, and SRMR indicated a well-fitting model (Table 5). Subtest loadings (Figure 4) on the general factor ranged from 0.49 (*Rover*) to 0.74 (*Riddles*). Group factors explained between 6% (*Learning/Glr*) and 11% (combined Gf/Gc factor) of the common variance. For group factors, ω_HS_ ranged from 0.23 (*Learning/Glr*) to 0.33 (*Sequential/Gsm*) (Table 8), whereas ω_H_ was 0.81 for the general factor.

Final model comparison: Comparing all models showed the highest Akaike weight for model 4b (*w_i_* = 0.998), followed by models 2, 2f, and 5 (*w_i_* = 0.001). Thus, a four-factorial, second-order structure with a cross-loading of *Story Completion* on *Knowledge/Gc* emerged as the best model.

### 3.3. Confirmatory Factor Analyses of Core Subtests (Without Time Points)

**Figure 5:**
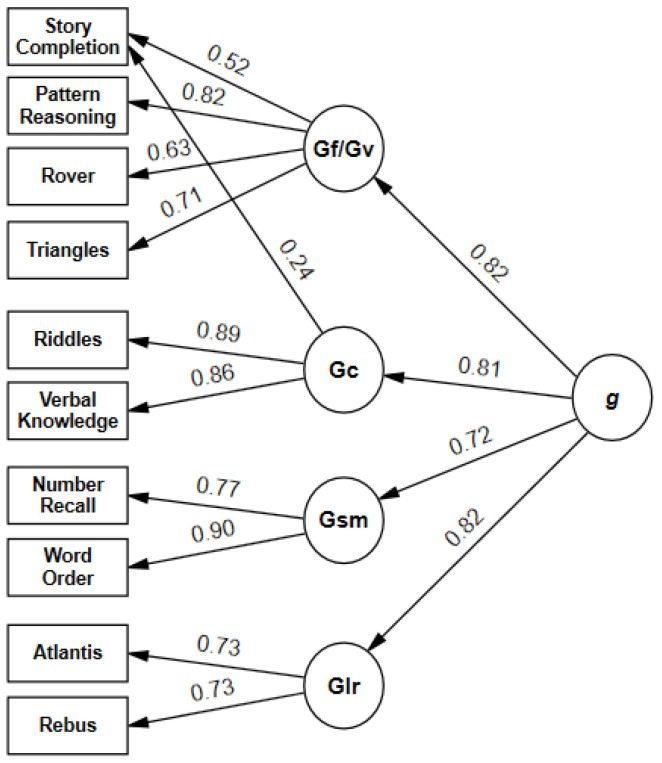
Overall best-fitting model (4b without time points) of the KABC-II core subtests. χ^2^ = 42.19, *df* = 31, *p* = 0.069, CFI = 0.996, RMSEA = 0.025, SRMR = 0.021.

CFAs of core subtests without time points were calculated for models 2, 2e, 3, 4, 4b, and 5. Global fit statistics for these models and ΔAIC values for the comparison with models with time points are shown in Table 9. All models showed an adequate fit according to CFI, RMSEA, and SRMR. Model 4b (Figure 5) was the only model with a non-significant χ^2^ test (*p* = 0.069) and was favored by Akaike weights (*w_i_* = 0.972), followed by model 2e (*w_i_* = 0.025) and model 3 (*w_i_* = 0.003). For each pairwise comparison of models with and without time points, ΔAIC (≥8.941) indicated superiority of models without time points (Table 9). An additional comparison of all models with and without time points confirmed the superiority of model 4b without time points (*w_i_* = 0.961).

## 4. Discussion

Published data on the factorial validity of KABC-II at ages 7 to 12 mostly relied on the KABC-II standardization samples and—except for analyses presented in the manuals and by McGill (2020)—did not exactly adhere to the structure of the KABC-II core subtests. Results of available studies raised some concerns about the adequacy of the factor structure, e.g., casting doubts on separating *Planning/Gf* and *Simultaneous/Gv*. This study closes a gap in the research on the factorial validity of the KABC-II by providing the first independent evaluation of the structure of core subtests in a clinical sample.

### 4.1. Standard Higher-Order Model of KABC-II Subtests

According to our criteria for the evaluation of global model fit, the standard higher-order model did not prove fully adequate when subtests with time points were included. While CFI and SRMR indicated an acceptable fit, RSMEA surpassed the cutoff. Discrepancies between different indices are not a rare occurrence in CFA and need not be related to model misspecification (Lai and Green 2016). McNeish et al. (2018) demonstrated that RMSEA values above common cutoff criteria can indicate an acceptable fit when factor loadings are high. Additionally, based on the more lenient cutoffs for RSMEA proposed by some authors (e.g., MacCallum et al. 1996) or for combinations of CFI and RSMEA (Hair et al. 2014), the model fit of the standard test structure might be considered acceptable. In summary, there was no clear indication of global model misfit.

However, global fit is not sufficient for a thorough evaluation of the KABC-II factor structure. AVE surpassed the threshold of 0.50 for all scales, although only minimally for *Simultaneous/Gv* and *Learning/Glr*. As in previous research, *Planning/Gf* and g were almost indistinguishable (λ = 0.96), indicating the redundancy of this factor. *Planning/Gf* and *Simultaneous/Gv* were highly intercorrelated, challenging the assumption that these factors can be meaningfully interpreted as measuring different constructs. The strong association between these factors replicates findings from the US and the German standardization samples and from McGill (2020).

Decomposed variance estimates show that on average, 41% of the total subtest variance was accounted for by the second-order factor, 21% by the first-order factor, and 38% by uniqueness (specificity and measurement error). Variance accounted for by *Planning/Gf* was negligible, whereas *Sequential/Gsm* accounted for more variance than the general factor.

Due to the multifaceted nature of several subtests, alternative models based on CHC theory could be generated. As in previous research, significant cross-loadings were found that aligned with the classification of narrow CHC abilities. *Pattern Reasoning* was more closely related to *Simultaneous/Gv* (λ = 0.55) than to *Planning/Gf* (λ = 0.32), leaving only *Story Completion* with a strong loading on *Planning/Gf*. A loading of *Story Completion* on *Knowledge/Gc* (λ = 0.19) vanished when both cross-loadings of *Pattern Reasoning* and *Story Completion* were allowed, simultaneously. Thus, model 2e emerged as the best of all 5-factorial models, underscoring the ambiguous character of *Pattern Reasoning*.

These results from 5-factorial higher-order models suggested that 4-factorial models, combining subtests of *Planning/Gf* and *Simultaneous/Gv*, might offer a better representation of the data. Indeed, these models were superior to the structure proposed by the test authors. All global fit indices showed that model 4 fit the data very well. The combined Gf/Gc factor and the general factor were less closely related (λ = 0.84) than *Planning/Gf* and the general factor in 5-factorial models. AVE was acceptable for all scales and ω was >0.80, except for *Learning/Glr*. Finally, model 4b, with a cross-loading of *Story Completion* on *Knowledge/Gc,* was the best of all the models with time points.

### 4.2. Bifactor vs. Higher-Order Structure Models

Fit indices for a classical bifactor model that does not allow cross-loadings (Zhang et al. 2021) and the standard higher-order model were identical. Both models demonstrated the importance of the general factor and led to identical conclusions in terms of variance accounted for by the general factor, and respectively, group factors, and uniqueness. For the four-factorial solution, the bifactor model showed excellent fit and was favored by ΔAIC compared to the higher-order model, but not compared to the higher-order model allowing *Story Completion* to load on *Knowledge/Gc*. Neither of the bifactor models demonstrated an ideal bifactor structure. Group factors lacked convergent validity, rendering their interpretation almost impossible. There was limited common variance between subtests when the general factor was accounted for.

There is an ongoing scholarly debate about whether bifactor or higher-order models are more adequate representations of the structure of multidimensional intelligence test batteries (e.g., Cucina and Byle 2017; Decker 2021; Dombrowski et al. 2021). From a theoretical perspective, both models differ in their assumptions on the relation between subtests and general intelligence (direct vs. mediated by broad abilities; see Keith and Reynolds 2018, for a comprehensive discussion), while some authors have pointed out communalities (Brunner et al. 2012; Gignac and Kretzschmar 2017). However, we hold that so far, this debate is of limited relevance for the clinical use of the KABC-II (see Renner et al. 2022). Unlike group factors in bifactor models, standardized scales of the KABC-II do not represent constructs that are uncorrelated with intelligence. Thus, a higher-order model is more in line with the test structure of the KABC-II. In clinical practice, test interpretation relies on standard scores provided in the manual. Standard scores for latent group factors are not available, and there is a complete lack of data on divergent, convergent, prognostic, and known-groups’ validity of group factors. However, the bifactor models of the KABC-II warn test users against interpreting scales as pure measures of specific constructs and against disregarding the influence of the general factor.

In analyses of the Wechsler Intelligence Scale for Children-V (WISC-V; Wechsler 2014) and its international adaptations, proponents of bifactor models have argued that clinicians should refrain from interpretation of subscales (e.g., Canivez et al. 2021; Dombrowski et al. 2018; Pauls and Daseking 2021). From a clinical perspective, we should like to add a cautionary note to this conclusion. In the case of significant profile heterogeneity, global IQ scores may not adequately represent cognitive functioning. Dissociation of cognitive abilities is obviously possible and common in children with developmental disorders and disabilities, as demonstrated by research on genetic syndromes (e.g., Williams syndrome; Miezah et al. 2021), neurological diseases (e.g., Landau-Kleffner-syndrome; Riccio et al. 2017), or autism spectrum disorder (Takayanagi et al. 2022). A cognitive test should be able to assess these dissociations because they are highly relevant for everyday functioning and planning of interventions. Of course, this presupposes that subscales represent these cognitive abilities specifically, rather than measuring a mixture of various intelligence factors.

### 4.3. Effects of Time Points

Rewarding speed introduces an additional component in two of five scales of the KABC-II. In terms of CHC theory, the broad ability *Processing Speed (Gs)* influences the results of some subtests but is not explicitly considered in the theoretical model and the test structure. Without time points, an acceptable fit was found for the standard test structure according to all fit indices. All models based on subtests without bonus points for rapid correct responses provided a better fit to the data than models with time points (ΔAIC ≥ 8.9). Again, allowing cross-loadings (models 2e and 4b) substantially improved the model fit.

The manuals of the KABC-II provide norms for tests without time points, but data on the reliability and validity are limited to subtests with time points. The reanalysis of Reynolds et al. (2007) and our results suggest that test users need not worry that calculating standard scores based on subtests with time points compromises the factorial validity of the KABC-II. We recommend that the effects of using time points should be considered in future psychometric studies (see Gernsbacher et al. 2020, for a comprehensive discussion of time-limited tests).

### 4.4. Limitations

Our results were based on a highly selected sample. Children had to be referred to a SPC by a pediatrician or general practitioner and intelligence testing had to be considered important by the SPC team. Referral questions, common institutional practices, specifics of the case (e.g., limitations in verbal or motor skills), and preferences of the examiner influenced the decision to use the KABC-II. The effects of this selection process remain unclear. Age of participants was not equally distributed over the total age range studied. Typically, for data collected in SPCs, males were overrepresented (e.g., Lüdeke et al. 2015; Renner et al. 2019). Therefore, our study does not allow generalization of findings to other clinical settings or the general population. Accordingly, we did not aim at estimating population parameters but instead intended to explore whether the data on factorial validity presented in the manuals of the KABC-II were generalizable to a clinical dataset.

Only core subtests could be included in our analyses. In the SPCs participating in this study, supplementary subtests were rarely used, probably because of time constraints and the need to avoid lengthy testing in children with limited attention and motivation. Thus, each scale of the KABC-II was represented by only two manifest variables, although a minimum of three indicators for each latent factor is preferable (Gignac and Kretzschmar 2017; Kline 2016). On the other hand, including all supplementary subtests would not have corresponded to the standard test structure of the KABC-II. Results of the re-analyses of the US standardization sample with all subtests (Reynolds et al. 2007) converged with our findings (e.g., *Pattern Reasoning* measuring multiple abilities, effects of time points).

Factor structures may differ for different age ranges. We aligned our analyses with the age range of confirmatory factor analyses reported in the manuals of the KABC-II. Nevertheless, more differentiated analyses (e.g., ages 7 to 8, etc.) might provide additional insight on the factor structure of the KABC-II.

A reviewer pointed out that our data (collected over a 6-year period) may have been affected by the Flynn effect. As research indicates that the Flynn effect has come to a standstill in Germany (Pietschnig et al. 2021), we did not assume a strong effect. However, there is some evidence that stratum II factors may be differentially affected by the Flynn effect (Lazaridis et al. 2022). Therefore, we cannot rule out the possibility that the correlations underlying the analyses were influenced by secular trends.

We evaluated several alternative factor models, mainly based on CHC theoretical classifications of subtests. In the age range studied, previous research did not suggest important additional hypotheses. We cannot exclude that other theoretical perspectives or statistical methods (e.g., exploratory bifactor analysis; Jennrich and Bentler 2011) might instigate further meaningful modifications. We refrained from using modification indices to improve the model fit without defensible theoretical arguments (see MacCallum et al. 1992; Tomarken and Waller 2003) and may have missed better representations of the data.

## 5. Conclusions

The authors of the KABC-II aimed to construct subtests and scales that measure specific intelligence factors, incorporate other abilities, and allow the derivation of a global intelligence score. Previous research and our results indicate that this intention and its realization are partly incompatible with a clear factorial structure. We suggest that the following key findings of this study should be considered in clinical practice when applying and interpreting the KABC-II:

- Our data showed that the scales of the KABC-II cannot be interpreted as dimensions independent of the general factor. Therefore, focusing mainly on the interpretation of scales and disregarding the influence of general intelligence on all scales is not recommended. At the same time, a general factor model that would support an interpretive strategy based solely on the total score was inferior to four- and five-factorial solutions.
- As in previous research, the distinction between *Planning/Gf* and *Simultaneous/Gv* is questionable. These scales seem to measure both visual and fluid abilities. Consequently, we caution against interpreting normative and intraindividual strengths and weaknesses in these scales as strong indicators of strengths and weaknesses in fluid intelligence, and respectively, visual processing. Accurate differentiation of fluid and visual abilities may require the use of additional tests that provide a purer measure of these intelligence factors.
- The strong additional loading of *Pattern Reasoning* on *Simultaneous/Gv* precludes an unequivocal interpretation of this subtest as measuring *Planning/Gf*. The cross-loading between *Story Completion* and *Knowledge/Gc* points to the influence of verbal processes in this subtest.
- Some subtests, notably *Rover*, *Number Recall*, and *Atlantis*, showed a large portion of unique variance. When used separately or as part of a cross-battery assessment, they should not be interpreted as strong measures of general intelligence or the presumed CHC factors.

We suggest that future development of intelligence test batteries should be guided by a systematic and thorough content analysis of test formats, linked to a clearly articulated theoretical basis. If the intention of a test is to measure specific abilities, it is important to develop unidimensional (sub-)tests that measure well-defined constructs (Canivez et al. 2021).

The importance of factorial validity for test interpretation is evident. However, it is not sufficient for responsible test use. So far, only a few studies (e.g., Benson et al. 2016; Irblich et al. 2020; Scheiber 2016; Scheiber and Kaufman 2015) have addressed other aspects of the validity, reliability, and fairness of the KABC-II and the interpretive strategy proposed by the publisher. We hope that future research will place more emphasis on these issues.

## Figures and Tables

**Table 1:** KABC-II core subtests and scales for 7- to 12-year-olds.

Scale Subtest	CHC Narrow Abilities Measured
Planning/Fluid Reasoning (Gf)	
Pattern Reasoning	Gf: Induction Gv: Visualization
Story Completion	Gf: Induction Gf: General Sequential Reasoning Gc: General Information Gv: Visualization
Simultaneous Processing/Visual Processing (Gv)	
Rover	Gv: Spatial Scanning Gf: General Sequential Reasoning Gq: Math Achievement
Triangles	Gv: Spatial Relations Gv: Visualization
Crystallized Ability (Gc)	
Riddles	Gc: Lexical Knowledge Gc: Language Development Gf: General Sequential Reasoning
Verbal Knowledge	Gc: Lexical Knowledge Gc: General Information
Sequential Processing/Short-Term Memory (Gsm)	
Number Recall	Gsm: Memory Span
Word Order	Gsm: Memory Span (without color interference) Gsm: Working Memory (with color interference)
Learning Ability/Long-Term Storage and Retrieval (Glr)	
Atlantis	Glr: Associative Memory
Rebus	Glr: Associative Memory

Note. According to Kaufman and Kaufman (2004a).

**Table 2:** Demographic characteristics of participants and the most common diagnoses.

Variable	*n* (%)
Age	
7;0–7;11	154 (24.6%)
8;0–8;11	168 (26.8%)
9;0–9;11	131 (20.9%)
10;0–10;11	85 (13.6%)
11;0–11;11	57 (9.1%)
12;0–12;11	32 (5.1%)
Sex	
Male	425 (67.8%)
Female	202 (32.2%)
Family structure	
Two-parent family	411 (65.6%)
Single-parent family	121 (19.3%)
Step-family	59 (9.4%)
Foster and residential care	31 (4.9%)
Other/unknown	5 (0.8%)
Migration	
None	466 (74.3%)
Parents only	124 (19.8%)
Child	25 (4.0%)
Other/unknown	12 (1.9%)
Most common psychological diagnoses (ICD-10, Chapter 5)	
Specific developmental disorders of scholastic skills (F81.x)	305 (48.6%)
Attention-deficit hyperactivity disorders (F90.x)	156 (24.9%)
Specific developmental disorders of speech and language (F80.x)	149 (23.8)
Emotional disorders with onset specific to childhood (F93.x)	116 (18.5%)
Other/Unspecified disorders of psychological development (F88.x, F89.x)	110 (17.5%)
Other behavioral and emotional disorders (F98.x)	103 (16.4%)
Conduct disorders (F91.x)	50 (8.0%)
Reaction to severe stress, and adjustment disorders (F43.x)	47 (7.5%)
Intellectual disabilities (F7x.x)	35 (5.6%)
Most common somatic diagnoses (ICD-10)	
Congenital malformations, deformations, and chromosomal abnormalities (Q00–Q99)	79 (12.6%)
Diseases of the nervous system (G00–G99)	65 (10.4%)
Symptoms, signs, and abnormal clinical and laboratory findings, not elsewhere classified (R00–R99)	63 (10.0%)
Endocrine, nutritional, and metabolic diseases (E00–E99)	47 (7.5%)
Diseases of the eye (H00–H59)	39 (6.2%)
Certain conditions originating in the perinatal period (P00–P96)	26 (4.1%)

Note. Multiple diagnoses per participant were possible.

**Table 3:** Overview of KABC-II subtest configurations for CFA models.

Model		SC	PR	ROV	TRI	RID	VK	NR	WO	ATL	REB
1	unidimensional	*g*	*g*	*g*	*g*	*g*	*g*	*g*	*g*	*g*	*g*
2	second-order	Gf	Gf	Gv	Gv	Gc	Gc	Gsm	Gsm	Glr	Glr
2a	second-order	Gf	Gf	Gv	Gv	Gc + Gf	Gc	Gsm	Gsm	Glr	Glr
2b	second-order	Gf + Gc	Gf	Gv	Gv	Gc	Gc	Gsm	Gsm	Glr	Glr
2c	second-order	Gf + Gv	Gf	Gv	Gv	Gc	Gc	Gsm	Gsm	Glr	Glr
2d	second-order	Gf	Gf	Gv + Gf	Gv	Gc	Gc	Gsm	Gsm	Glr	Glr
2e	second-order	Gf	Gf + Gv	Gv	Gv	Gc	Gc	Gsm	Gsm	Glr	Glr
2f	second-order	Gf + Gv	Gf + Gv	Gv	Gv	Gc	Gc	Gsm	Gsm	Glr	Glr
3	bifactor	*g*, Gf	*g*, Gf	*g*, Gv	*g*, Gv	*g*, Gc	*g*, Gc	*g*, Gsm	*g*, Gsm	*g*, Glr	*g*, Glr
4	second-order	Gf/Gv	Gf/Gv	Gf/Gv	Gf/Gv	Gc	Gc	Gsm	Gsm	Glr	Glr
4a	second-order	Gf/Gv	Gf/Gv	Gf/Gv	Gf/Gv	Gc, Gf/Gv	Gc	Gsm	Gsm	Glr	Glr
4b	second-order	Gf/Gv, Gc	Gf/Gv	Gf/Gv	Gf/Gv	Gc	Gc	Gsm	Gsm	Glr	Glr
5	bifactor	*g*, Gf/Gv	*g*, Gf/Gv	*g*, Gf/Gv	*g*, Gf/Gv	*g*, Gc	*g*, Gc	*g*, Gsm	*g*, Gsm	g, Glr	g, Glr

Note. SC = *Story Completion*; PR = *Pattern Reasoning*; ROV = *Rover*; TRI = *Triangles*; RID = *Riddles*; VK = *Verbal Knowledge*, NR = *Number Recall*, WO = *Word Order*; ATL = *Atlantis*; REB = *Rebus*; g = general factor of intelligence; Gf = *Planning/Gf*; Gv = *Simultaneous/Gv*; Gc = *Knowledge/Gc*; Gsm = *Sequential/Gsm*; Glr = *Learning/Glr*; Gf/Gv = combined factor *Planning/Gf* and *Simultaneous/Gv*.

**Table 4:** Confirmatory factor analysis fit statistics for KABC-II core subtest CHC configurations.

Model	χ^2^	*df*	*p*	CFI	RMSEA	90% CI RMSEA	SRMR	AIC	ΔAIC	*w_i_* AIC
1 *g*-factor	609.238	35	0.000	0.795	0.162	[0.151, 0.173]	0.076	649.238	548.107	0.000
2 second-order	106.166	30	0.000	0.973	0.064	[0.051, 0.077]	0.038	156.166	55.035	0.000
2a (Gf -> RID)	Inadmissible solution									
2b (Gc -> SC)	97.873	29	0.000	0.975	0.062	[0.048, 0.075]	0.038	149.873	48.742	0.000
2c (Gv -> SC)	106.152	29	0.000	0.973	0.065	[0.052, 0.079]	0.039	158.152	57.021	0.000
2d (Gf -> ROV)	Inadmissible solution									
2e (Gv -> PR)	64.498	29	0.002	0.987	0.044	[0.030, 0.059]	0.027	116.498	15.367	0.001
2f (Gc -> SC, Gv -> PR)	106.152	29	0.001	0.988	0.044	[0.030, 0.059]	0.028	116.388	15.257	0.001
3 bifactor	64.498	29	0.000	0.973	0.064	[0.051, 0.077]	0.038	156.166	55.035	0.000
4 second-order (Gf/Gv)	72.868	31	0.000	0.985	0.046	[0.033, 0.060]	0.029	120.868	19.737	0.000
4a (Gf/Gv -> RID)	72.468	30	0.001	0.985	0.048	[0.034, 0.062]	0.028	122.868	21.337	0.000
4b (Gc -> SC)	51.131	30	0.025	0.992	0.034	[0.017, 0.049]	0.022	101.131	0.000	0.998
5 Bifactor (Gf/Gv)	66.487	31	0.003	0.987	0.043	[0.029, 0.057]	0.026	114.487	13.356	0.001

Note. CFI = comparative fit index; RMSEA = root mean square error of approximation; CI = confidence interval; SRMR = standardized root mean square residual; AIC = Akaike information criterion; ΔAIC = difference from the lowest AIC of all models; *w_i_* AIC = Akaike weight; Gf = *Planning/Gf*; RID = *Riddles*; Gc = *Knowledge/Gc*; SC = *Story Completion*; Gv = *Simultaneous/Gv*; ROV *= Rover*; PR = *Pattern Reasoning*; Gf/Gv = combined *Planning/Gf* and *Simultaneous/Gv* factor. *p*-Values are based on the Bollen–Stine bootstrap method.

**Table 5:** Loadings of CHC core subtests on the general factor in unidimensional measurement models.

	Time Points	No Time Points
Story Completion	0.70	0.69
Pattern Reasoning	0.74	0.73
Rover	0.56	0.55
Triangles	0.64	0.63
Riddles	0.77	0.77
Verbal Knowledge	0.75	0.76
Number Recall	0.57	0.57
Word Order	0.64	0.64
Atlantis	0.58	0.58
Rebus	0.59	0.58

Note. All loadings are significant at *p* < 0.001.

**Table 6:** Second-order models: coefficient omega (ω) and average variance extracted (AVE).

Factor	Model 2		Model 4	
	ω	AVE	ω	AVE
Planning/Gf	0.75	0.60	0.81	0.53
Simultaneous/Gv	0.67	0.51		
Knowledge/Gc	0.87	0.77	0.87	0.77
Sequential/Gsm	0.82	0.70	0.82	0.70
Learning/Glr	0.69	0.53	0.69	0.52

Note. ω = coefficient omega, AVE = average variance extracted.

**Table 7:** Five-factorial bifactor model of KABC-II core subtests: factor loadings and sources of variance.

Subtest	General		Gf		Gv		Gc		Gsm		Glr		Unique Var
	λ	Var	λ	Var	λ	Var	λ	Var	λ	Var	λ	Var	
Story Completion	0.72	0.52	0.19	0.04									0.45
Pattern Reasoning	0.78	0.61	0.22	0.05									0.35
Rover	0.57	0.32			0.32	0.10							0.58
Triangles	0.68	0.46			0.37	0.14							0.40
Riddles	0.69	0.48					0.55	0.31					0.22
Verbal Knowledge	0.67	0.45					0.56	0.31					0.24
Number Recall	0.51	0.26							0.61	0.38			0.36
Word Order	0.59	0.35							0.63	0.40			0.26
Atlantis	0.55	0.30									0.46	0.21	0.49
Rebus	0.58	0.33									0.47	0.22	0.45
ECV	0.66		0.01		0.04		0.10		0.13		0.07		
ω/ω_S_	0.92		0.75		0.67		0.87		0.82		0.69		
ω_H_/ω_HS_	0.83		0.05		0.16		0.35		0.46		0.28		

Note. Gf = *Planning/Gf*; Gv = *Simultaneous Processing/Gv*; Gc = *Knowledge/Gc*; Gsm = *Sequential Processing/Gsm*; Glr = *Learning/Glr*; λ = standardized factor loading; Var = % variance explained; *h²* = communality; ECV = explained common variance; ω = coefficient omega; ω_S_ = coefficient omega subscale; ω_H_ = coefficient omega hierarchical; ω_HS_ = coefficient omega hierarchical subscale.

**Table 8:** Four-factorial bifactor model of KABC-II core subtests: factor loadings and sources of variance.

Subtest	General		Gf/Gv		Gc		Gsm		Glr		Unique Var
	λ	Var	λ	Var	λ	Var	λ	Var	λ	Var	
Story Completion	0.64	0.41	0.37	0.14							0.45
Pattern Reasoning	0.69	0.48	0.42	0.18							0.34
Rover	0.49	0.24	0.37	0.14							0.62
Triangles	0.58	0.34	0.44	0.19							0.47
Riddles	0.74	0.55			0.50	0.25					0.21
Verbal Knowledge	0.71	0.50			0.50	0.25					0.25
Number Recall	0.55	0.30					0.59	0.35			0.35
Word Order	0.64	0.41					0.44	0.19			0.40
Atlantis	0.59	0.35							0.42	0.18	0.48
Rebus	0.60	0.36							0.42	0.18	0.46
ECV	0.66		0.11		0.08		0.09		0.06		
ω/ω_S_	0.92		0.82		0.87		0.77		0.69		
ω_H_/ω_HS_	0.81		0.25		0.28		0.33		0.23		

Note. Gf/Gv = a combined *Planning/Gf* and *Simultaneous/Gv* factor; Gc = *Knowledge/Gc*; Gsm = *Sequential/Gsm*; Glr = *Learning/Glr*; λ = standardized factor loading; Var = % variance explained; *h²* = communality; ECV = explained common variance; ω = coefficient omega; ω_S_ = coefficient omega subscale; ω_H_ = coefficient omega hierarchical; ω_HS_ = coefficient omega hierarchical subscale.

**Table 9:** Confirmatory factor analysis fit statistics for the KABC-II core subtest CHC configurations without time points.

Model	χ^2^	*df*	*p*	CFI	RMSEA	90% CI RMSEA	SRMR	AIC	ΔAIC	*w_i_* AIC	ΔAIC Time Points ^a^
2 second-order	90.50	30	<.001	0.978	0.057	[0.044, 0.070]	0.035	140.495	48.31	0.000	15.671
2e (Gv → PR)	47.53	29	.016	0.993	0.032	[0.014, 0.048]	0.023	99.526	7.34	0.025	16.972
3 bifactor	90.50	30	<.001	0.978	0.057	[0.044, 0.070]	0.035	140.495	48.31	0.000	15.671
4 second-order (Gf/Gv)	61.81	31	.001	0.989	0.040	[0.025, 0.054]	0.028	109.807	17.62	0.000	11.061
4b (Gc → SC)	42.19	30	.069	0.996	0.025	[0.026, 0.055]	0.021	92.190	0.00	0.972	8.941
5 bifactor (Gf/Gv)	55.79	31	.004	0.991	0.036	[0.020, 0.051]	0.025	103.794	11.60	0.003	10.693

Note. CFI = comparative fit index; RMSEA = root mean square error of approximation; CI = confidence interval; SRMR = standardized root mean square residual; AIC = Akaike information criterion; ΔAIC = difference from the lowest AIC of all models; *w_i_* AIC = Akaike weights; Gf = *Planning/Gf*; Gc = *Knowledge/Gc*; Gv = *Simultaneous/Gv*; RID = *Riddles*; SC = *Story Completion*; ROV *= Rover*; PR = *Pattern Reasoning. p*-Values are based on the Bollen–Stine bootstrap method. ^a^ Differences between AIC of the model without time points and the corresponding model with time points (see Table 4). Positive values favor models without time points.

## Data Availability

Data are available from the first named author upon reasonable request.

## Supplementary Materials

The following are available online: https://www.mdpi.com/article/10.3390/jintelligence11070148/s1. Table S1: Descriptive statistics for KABC-II subtests, scales, and global scales. Table S2: Intercorrelations of KABC-II core subtests. Table S3: Standard second-order CHC model: loadings of first-order factors on the general factor and implied correlations of first-order factors for core subtests with and without time points.
