# Supplementary material for: Factorial Validity of the German KABC-II at Ages 7 to 12 in a Clinical Sample: Four Factors Fit Better than Five
Source: J Intell. 2023 Jul 22;11(7):148. doi: 10.3390/jintelligence11070148 (PMC10381406; doi:10.3390/jintelligence11070148)
Supplement: Supplementary file 1 [file jintelligence-11-00148-s001.zip › jintelligence-2486596-supplementary.pdf]

Journal of
Intelligence

Article

Factorial Validity of the German KABC-II at Ages 7 to 12 in a Clinical Sample: Four Factors Fit Better than Five

Gerolf Renner [1,*], Anne Schroeder [2] and Dieter Irblich [3,†]

[1] Ludwigsburg University of Education, 71634 Ludwigsburg, Germany
[2] Werner Otto Institute, 22337 Hamburg, Germany
[2] formerly Social Pediatric Center kreuznacher diakonie, 55469 Simmern, Germany
* Correspondence: renner@ph-ludwigsburg.de
† Current address: Private Psychotherpeutic Practice, 55469 Simmern, Germany

Supplementary Materials

Table S1. Descriptive statistics for KABC-II subtests, scales, and global scales

Table S2. Intercorrelations of KABC-II core subtests

Table S3. Standard second order CHC model: Loadings of first-order factors on the general factor and implied correlations of first-order factors for core subtests with and without time points

Journal of Intelligence

Table S1. Descriptive statistics for KABC-II subtests, scales, and global scales

	Mean	SD	Skewness	Kurtosis
Story Completion	7.69	3.30	0.18	-0.50
Pattern Reasoning	8.74	2.96	0.12	0.47
Rover	8.91	3.34	0.14	-0.75
Triangles	7.52	2.86	-0.10	-0.16
Riddles	9.03	3.19	-0.29	0.01
Verbal Knowledge	8.39	3.16	0.06	0.20
Number Recall	7.58	2.97	0.15	-0.03
Word Order	8.50	3.06	0.25	0.06
Atlantis	9.01	3.05	-0.08	0.73
Rebus	8.10	3.01	0.08	-0.26
Planning/Gf	89.39	16.88	-0.13	0.24
Simultaneous/Gv	92.39	15.04	0.05	0.62
Knowledge/Gc	92.74	16.50	-0.30	1.34
Sequential/Gsm	88.68	15.75	-0.01	0.24
Learning/Glr	90.28	15.39	-0.14	0.08
Fluid-Crystallized Index	88.24	14.34	0.16	0.39
Mental Processing Index	87.75	14.33	0.14	0.37

Journal of *Intelligence*

MDPI

Table S2. Intercorrelations of KABC-II core subtests

	Story Completion		Pattern Reasoning		Rover	Triangles		Riddles	Verbal Knowledge	Number Recall	Word Order	Atlantis
	time points	no time points	time points	no time points		time points	no time points					
Story Completion (no tp)	.93											
Pattern Reasoning (tp)	.61	.58										
Pattern Reasoning (no tp)	.58	.58	.96									
Rover	.40	.41	.51	.53								
Triangles (tp)	.52	.49	.61	.59	.52							
Triangles (no tp)	.51	.50	.61	.59	.50	.95						
Riddles	.53	.52	.51	.52	.38	.42	.43					
Verbal Knowledge	.53	.52	.49	.50	.37	.44	.44	.77				
Number Recall	.33	.35	.40	.39	.32	.32	.32	.42	.37			
Word Order	.37	.39	.43	.42	.33	.33	.34	.50	.45	.69		
Atlantis	.42	.42	.39	.40	.26	.34	.34	.42	.41	.36	.43	
Rebus	.39	.40	.44	.42	.30	.40	.39	.40	.39	.31	.41	.52

Note. All correlations are significant with $p < .001$. tp = time points

Journal of Intelligence

MDPI

Table S3. Standard second order CHC model: Loadings of first-order factors on the general factor and implied correlations of first-order factors for core subtests with and without time points

Factor	g	Gf	Gv	Gc	Gsm	Glr
g	---	0.96	0.88	0.77	0.66	0.77
Gf	0.98	---	0.85	0.75	0.64	0.75
Gv	0.88	0.87	---	0.68	0.58	0.68
Gc	0.78	0.77	0.69	---	0.51	0.60
Gsm	0.67	0.66	0.59	0.52	---	0.51
Glr	0.77	0.76	0.68	0.61	0.52	---

Note. Values are based on model 2 with time points (above the diagonal) and without time points (below the diagonal).
